# A new genus and species of Labeonini (Teleostei: Cyprinidae) from the Pearl River in China

**DOI:** 10.1371/journal.pone.0199973

**Published:** 2018-07-06

**Authors:** Lan-Ping Zheng, You He, Jun-Xing Yang, Lun-Biao Wu

**Affiliations:** 1 State Key Laboratory of Genetic Resources and Evolution, Kunming Institute of Zoology, Chinese Academy of Sciences, Kunming, China; 2 Shanghai Synchrotron Radiation Facility, Shanghai Institute of Applied Physics, Chinese Academy of Sciences, Shanghai, China; 3 Fishery Bureau of Beiliu City, Beiliu, China; SOUTHWEST UNIVERSITY, CHINA

## Abstract

*Zuojiangia jingxiensis*, both a new genus and species, is described from the Pearl River in China. It is distinguished from all other genera and species of Labeonini by the unique combination of modified oromandibular structures and head skeleton: a well-developed, pendulous, and conspicuously arched rostral fold, with an entirely crenulated margin; prominent papillae densely covering the margin of the rostral fold and anterior part of the lower lip; long postlabial grooves, partitioning the lower lip into three parts; transverse branch of dentary longer than half the length of the longitudinal branch; stubby lateral process present at the anterolateral margin of the longitudinal branch of the dentary, close to the corner; in the upper jaw, the premaxilla bears a triangular ascending process tapering to a point; maxilla exhibits a pair of articular heads at the anterodorsal margin, and a distinct fingerlike descending process posterior to the medial articular head embracing the ascending process of the premaxilla.

## Introduction

The cyprinid tribe Labeonini exhibits a high degree of morphological modification in the oromandibular structures. More than 30 genera of Labeonini are currently recognized, most of which are distributed in China [1, 2]. Moreover, the species in the karst regions of southwestern China account for more than half of the genera and species of the Labeonini in China [2]. It can be seen that China exhibits a high species diversity of Labeonini. Recently, several new genera have been established in China, with their validity confirmed by molecular studies [2, 3]. The past results of molecular phylogeny in Labeonini were not consistent with those of morphological phylogeny, and the morphological characters do not have systematic significance but can be useful for taxonomy [4].

Guangxi Province is home to the karst regions of southern China, which are characterized by abundant limestone formations and underground rivers, as well as high biodiversity. Since 2000, eleven new species and four new genera of labeonins have been reported from Guangxi [4–14]. Three of the new species (*Pseudogyrinocheilus longisulcus*, *Cophecheilus bamen*, and *Rectoris longibarbus*) and one genus (*Cophecheilus*) are from the Jinxi area in Guangxi [11, 13, 15], a region known for great fish species diversity.

During examination of fish collections from Guangxi, an undescribed genus was discovered. Based on this examination, the oromandibular structure was described and the head skeleton was illustrated using X-ray microtomography. A combination of morphological characters distinguishes the new genus from all other known labeonine genera; hence, a new genus is required, which is described herein.

## Materials and methods

### Measurements and observations

Measurements and counts follow Chu and Chen [16] and Kottelat [17]. The methods used for counting the branched rays of the dorsal and anal fins and lateral line scales are described in Zheng et al. [12]. Vertebrae were counted from radiographs taken by a Digital Cabinet X-ray System (Milford, USA). Osteological characters were scanned using a MicroCT Skyscan 1176 (Bruker, Belgium) with 50 kV tube voltage, 0.3-degree rotation step, and 9-μm pixel resolution, and the cross-sections of each specimen were reconstructed using the associated software. Three-dimensional renderings were created, visualized, and manipulated using VG Studio Max (v2.1) software. By adjusting the gray value threshold, the structure of the bones remained while the soft tissues were virtually removed [18]. Examined specimens are in the collection of the Kunming Institute of Zoology (KIZ), Chinese Academy of Sciences and the Fishery Bureau of Du’an County (FBD). Abbreviations used in the text are: SL, standard length; HL, lateral head length. In addition, a morphological phylogenetic analysis was conducted with maximum likelihood (ML) approach using PAUP* 4.0b 10 [19].

We followed all guidelines of the Animal Ethics Committee of the Kunming Institute of Zoology, Chinese Academy of Sciences. All work was approved by the committee and did not involve any endangered species or protected areas (SMKX-2012019).

### Nomenclatural acts

The electronic edition of this article conforms to the requirements of the amended International Code of Zoological Nomenclature, and hence the new names contained herein are available under that Code from the electronic edition of this article. This published work and the nomenclatural acts it contains have been registered in ZooBank, the online registration system for the ICZN. The ZooBank LSIDs (Life Science Identifiers) can be resolved and the associated information viewed through any standard web browser by appending the LSID to the prefix “http://zoobank.org/”. The LSID for this publication is: urn:lsid:zoobank.org:pub: 56A190D5-AA22-4381-975B-414F97A29354. The electronic edition of this work was published in a journal with an ISSN, and has been archived and is available from the following digital repositories: PubMed Central, LOCKSS.

## Results

### *Zuojiangia*, new genus

urn:lsid:zoobank.org:act:340B0373-4D74-41C9-9E33-CA408B2DB9DB

**Fig 1:**
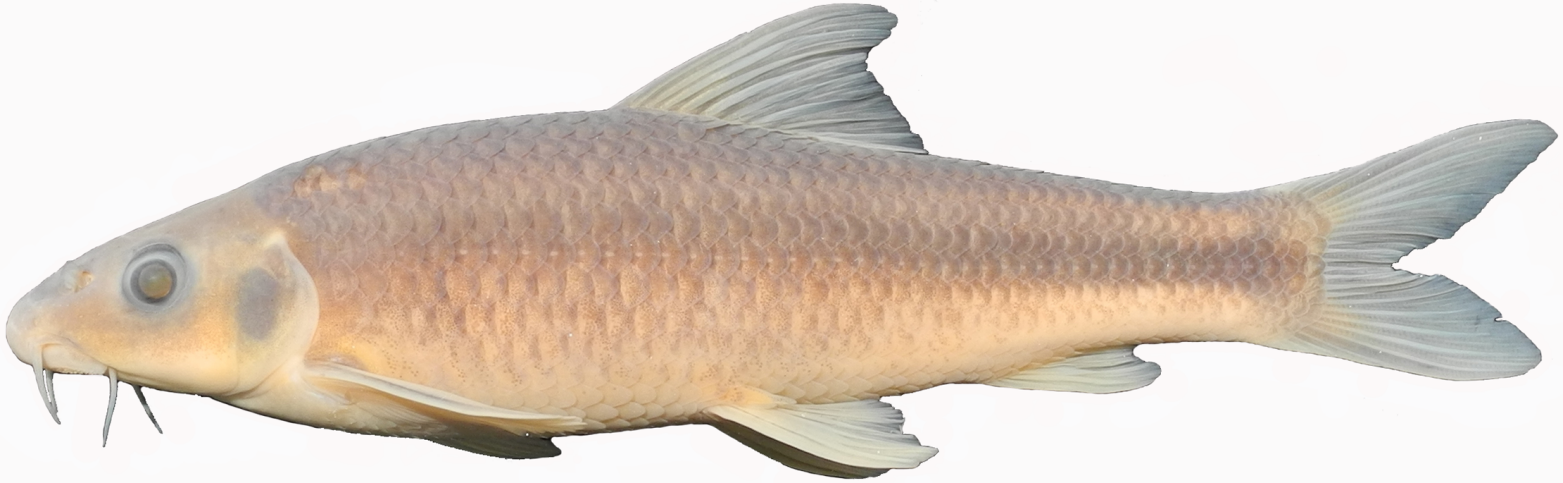
*Zuojiangia jingxiensis*, paratype, FBD 2010080053, 92.2 mm SL; China: Guangxi: Pearl River.

**Fig 2:**
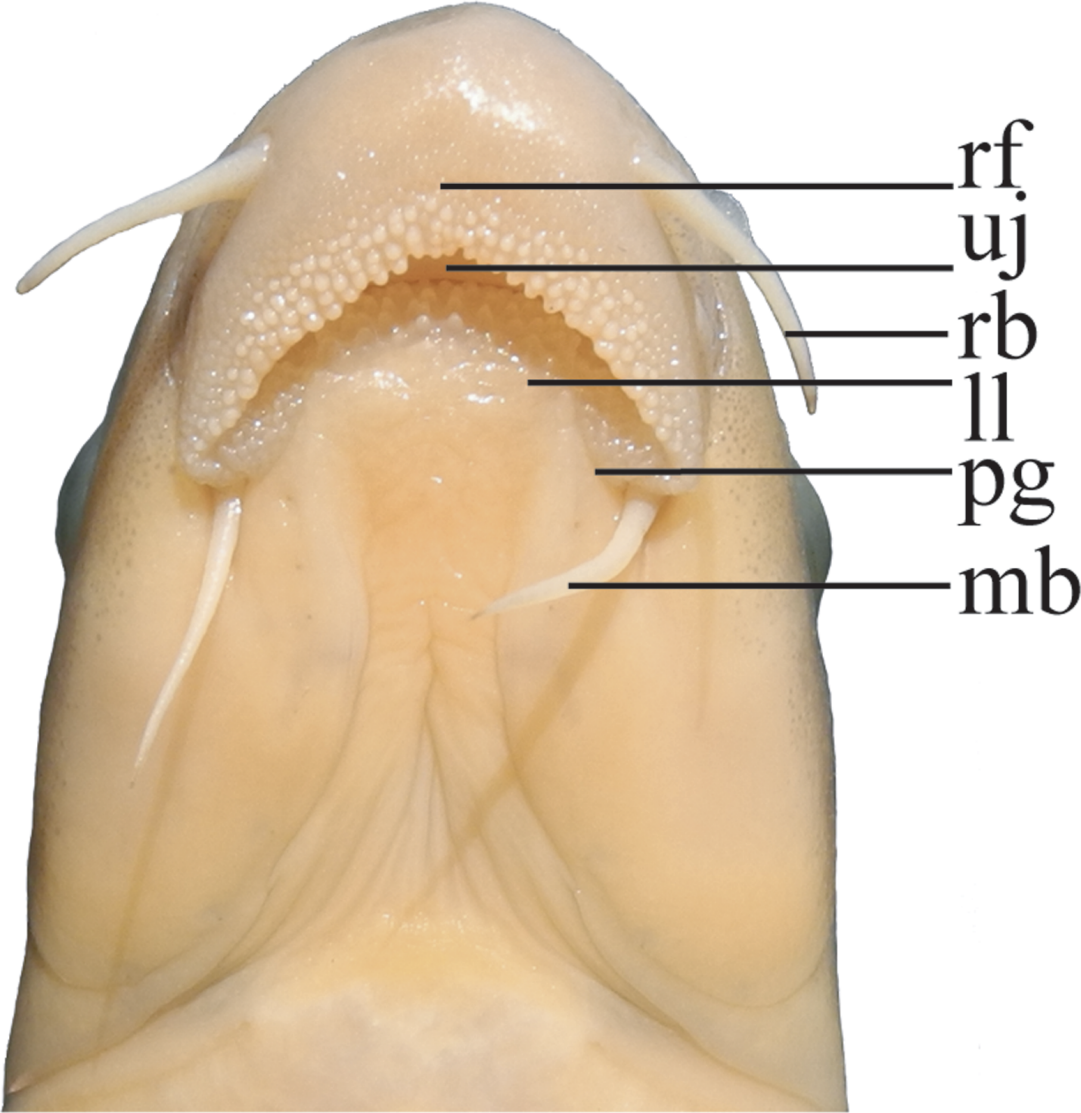
Ventral view of mouth of *Zuojiangia jingxiensis*, paratype, FBD 2010080053, 112.1 mm SL. Abbreviations, rf: rostral fold, uj: upper jaw, ll: lower lip, rb: rostral barbell, mb: maxillary barbels, pg: postlabial grooves.

**Fig 3:**
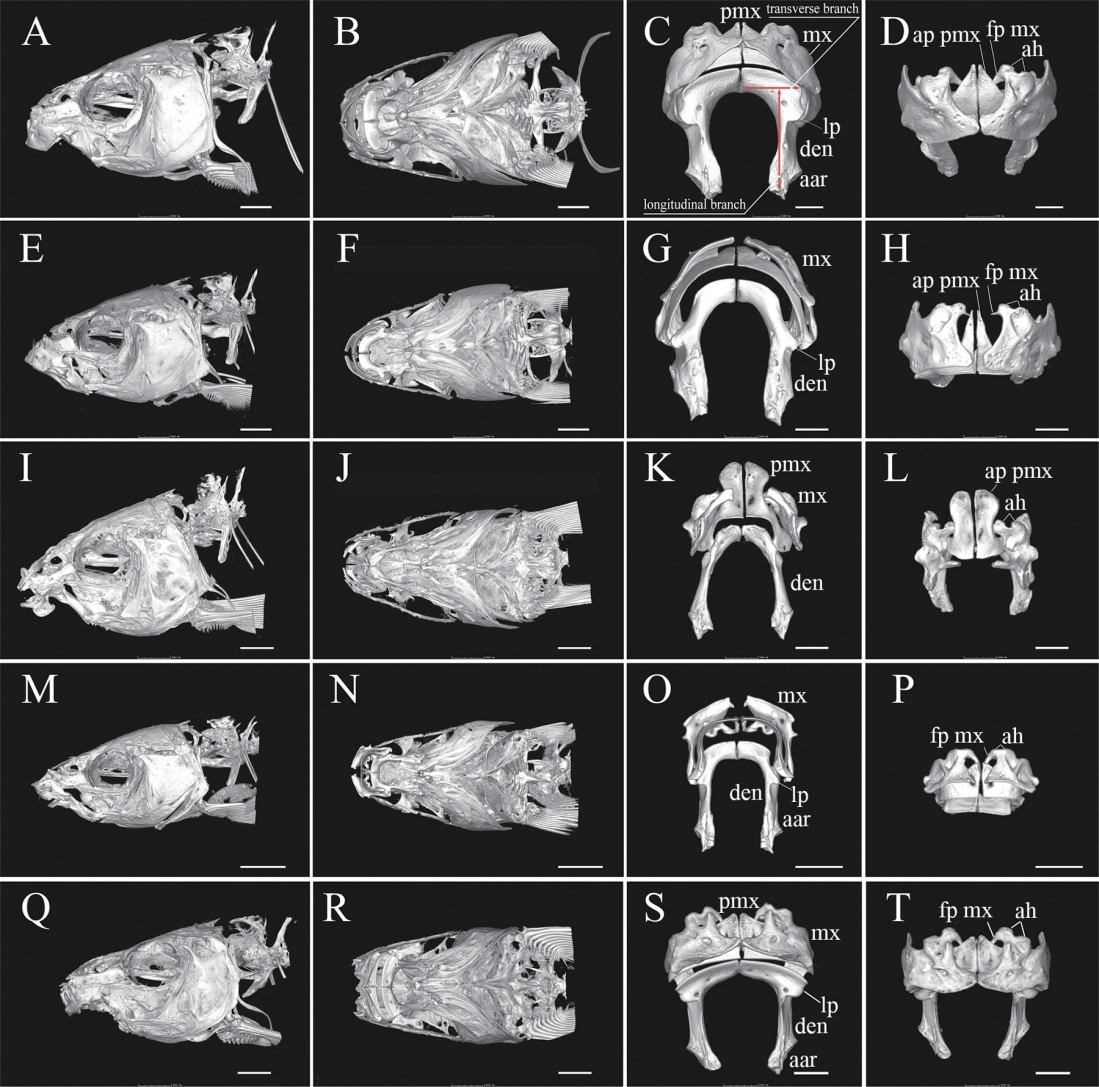
Head skeleton and jaw of *Zuojiangia jingxiensis* holotype, KIZ 2012003910 (A–D), *Cophecheilus bamen*, KIZ 2015001867 (E–H), *Prolixicheilus longisulcus*, KIZ 20100064 (I–L), *Parasinilabeo longibarbus*, KIZ 20100007 (M–P), and *Rectoris posehensis*, KIZ 20170170 (Q–T). Abbreviations, pmx: premaxilla, mx: maxilla, den: dentary, lp: lateral process, aar: anguloarticular, ah: articular head, fp mx: fingerlike process of maxilla, ap pmx: ascending process of premaxilla. Scale bar: 4.0 mm in Q, R; 3.5mm in A, B, E, F; 3.0 mm in I, J, M, N; 2.0 mm in S, T; 1.5 mm in C, D, G, H, K, L, O, P.

**Fig 4:**
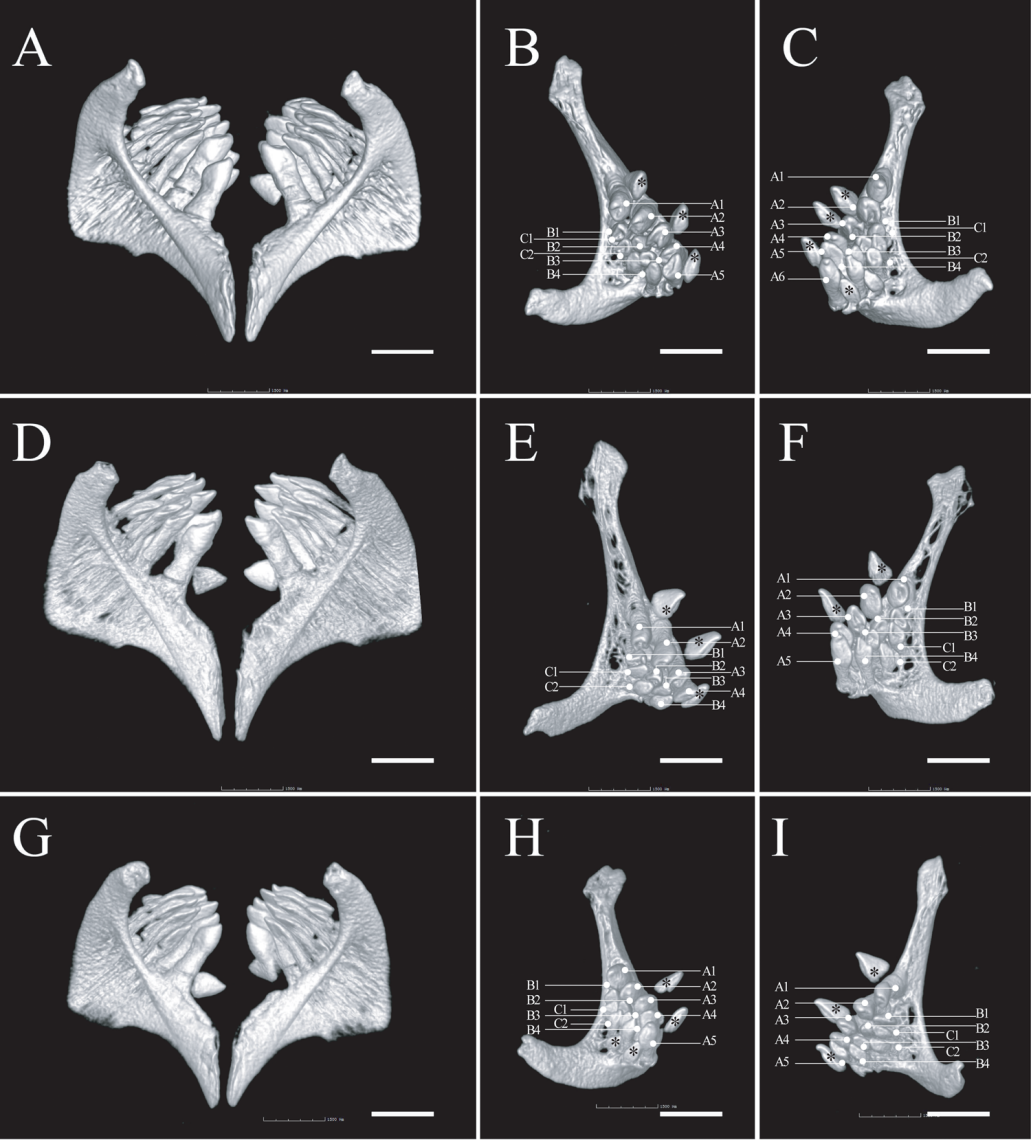
Pharyngeal dentition of *Zuojiangia jingxiensis* A–C, holotype, KIZ 2012003910; D–F, paratype, KIZ 2012003911; G–I, paratype, KIZ 2012005694. A, D, G, paired ceratobranchial 5 in anterior-oblique view. B, E, H, left ceratobranchial 5 in dorsal-oblique view. Scale bar: 1.5 mm. Abbreviations, “A”: main row of teeth; “B” medial secondary row, “C” most lateral secondary row, stars indicate replaced teeth.

Type species. ***Zuojiangia jingxiensis*** sp. nov. (Figs 1–4)

urn:lsid:zoobank.org:act:5152DC35-9FDC-4382-93AF-88E95396D0F8

#### Diagnosis

*Zuojiangia* is distinguished from all other genera of Labeonini by having a well-developed, pendulous and conspicuously arched rostral fold, with an entirely crenulated margin; prominent papillae densely covering the margin of the rostral fold and anterior part of the lower lip; and long postlabial grooves, partitioning the lower lip into three parts.

In the new genus, the left dentary is shaped like an inverted L in ventral view (Fig 3C, right dentary is a mirror image), with the transverse branch being longer than half the length of the longitudinal branch (Fig 3C). A stubby lateral process is present at the anterolateral margin of the longitudinal branch (Fig 3C, lp), close to the corner. In the upper jaw, the premaxilla bears a triangular ascending process tapering to a point (Fig 3D). The maxilla has a pair of articular heads at the anterodorsal margin, and a distinct fingerlike descending process posterior to the medial articular head embracing the ascending process of the premaxilla (Fig 3D, fp mx). There are three rows of teeth on the pharyngeal bone (= ceratobranchial 5, Fig 4). The teeth are coarsely compressed (Fig 4A, 4D and 4G). It is worth noting that the dentition formula was variable among the three examined specimens: 2.4.5–6.4.2 in holotype KIZ2012003910, 2.4.4–5.4.2 in paratype KIZ2012003911, and 2.4.5–5.4.2 in paratype KIZ2012005694. The main row on the right ceratobranchial 5 had six teeth in the holotype (Fig 4C), but four on the left side of one of the paratypes (Fig 4E).

#### Etymology

Named for Zuojiang River, where the type species was discovered. The gender is feminine.

#### Remarks

**Fig 5:**
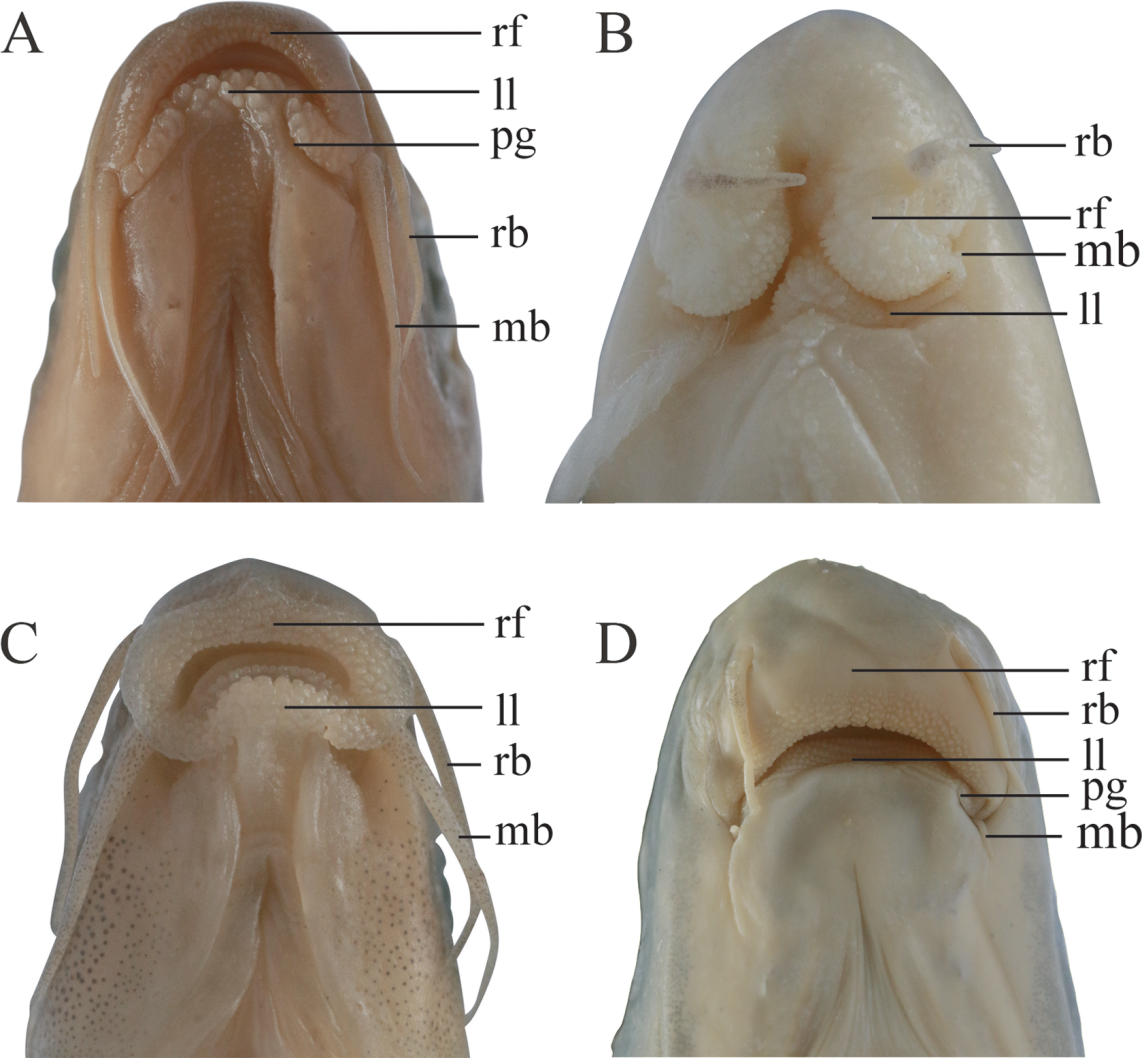
Oromandibular structures of *Cophecheilus bamen*, KIZ 2015001868, 87.0 mm SL (A), *Prolixicheilus longisulcus*, KIZ 20100064, 67.1 mm SL (B), *Parasinilabeo longibarbus*, KIZ 20100010, 50.3 mm SL (C), and *Rectoris posehensis*, KIZ 20170169, 120.0 mm SL (D). Abbreviations, rf: rostral fold, uj: upper jaw, ll: lower lip, rb: rostral barbell, mb: maxillary barbels, pg: postlabial grooves.

*Zuojiangia* is distinguished from all other genera of Labeonini except for *Cophecheilus*, *Prolixicheilus*, and *Parasinilabeo* *longibarbus* by having long postlabial grooves, complete rostral fold, and developed papillae covering the rostral fold and/or lower lip. Although *Zuojiangia* resembles the above three genera, it can be easily distinguished morphologically (Fig 5). It is differentiated from *Cophecheilus* by having no shallow arched, subdistal depression along the margin of the rostral cap (vs. present), margin of the rostral fold crenulated (vs. medially slightly furrowed but non-fimbriate), and 16–18 circumpeduncular scales (vs. 22–26). *Zuojiangia* differs from *Prolixicheilus* by the margin of the rostral fold crenulated (vs. smooth), rostral fold pendulous (vs. everted), posterior margin of lower lip not free, connected with chin (vs. free), long barbels, extending to eyes (vs. short, not reaching), and 43–46 lateral-line scales (vs. 40–42). The new genus is distinct from *P*. *longibarbus* by possessing an arched margin of the rostral fold (vs. crescentic), 43–46 lateral-line scales (vs. 37–40), 41–44 vertebrae (vs. 40), and medium sized body (94–114 mm SL vs. small, 44–53 mm SL).

***Zuojiangia jingxiensis* sp. nov.** (Figs 1 and 2; Table 1, S1 Table)

**Table 1:** Morphometric data for *Zuojiangia jingxiensis* SD = standard deviation.

	*Zuojiangia jingxiensis*, 5 paratypes and the holotype			
	Holotype	Range	Mean	SD
**Standard length (mm)**	112.1	92.2–113.9	103.3	-
**Percentage of SL**				
Body depth	24.1	20.8–25.1	23.6	1.54
Head length	21.4	21.4–23.9	22.4	1.07
Head depth	15.3	14.1–15.3	14.9	0.46
Head width	13.9	13.0–15.0	14.0	0.66
Dorsal-fin length	23.2	21.2–24.7	23.2	1.31
Pectoral-fin length	20.5	18.9–30.0	22.3	3.98
Pelvic-fin length	17.0	16.6–20.5	18.2	1.41
Anal-fin length	16.3	14.3–18.2	16.6	1.46
Caudal peduncle length	17.4	17.4–19.7	18.5	0.89
Caudal peduncle depth	10.9	10.4–12.6	11.5	0.76
Predorsal length	45.0	42.9–49.5	46.6	2.46
Prepectoral length	19.4	18.7–23.8	20.2	1.87
Prepelvic length	48.9	48.8–54.3	50.6	2.05
Preanal length	73.5	72.7–78.4	73.9	2.24
**Percentage of HL**				
Snout length	44.6	41.9–49.6	44.7	2.86
Head depth	71.3	61.7–71.3	66.4	3.56
Eye diameter	20.4	19.3–23.3	20.9	1.40
Interorbital width	44.6	42.3–46.8	43.8	1.67

#### Holotype

KIZ 2012003910, 112.1mm SL; China: Guangxi Province: Jingxi: Lutong Town: Biaoliang Village; Zuojiang River, a tributary of the Pearl River, 23° 9.193' N, 106°15.985' E, August 2010, J. Lan.

#### Paratypes

KIZ 2012003909, 3911–3912, 5694, FBD 2010080053, five specimens, 92.2–113.9 mm SL; collected with holotype.

#### Diagnosis

See generic diagnosis for characters distinguishing the species from all other labeonins.

#### Description

Morphometric data are listed in Table 1. Body rounded, and caudal peduncle compressed. Highest point of body usually in front of dorsal-fin origin. Head rounded, depth greater than width. Snout moderately rounded, longer than postorbital length. Eye moderately large, in posterior half of head, close to dorsal profile. Largest measured specimen 113.9 mm SL. Three rows of pharyngeal teeth (Fig 4).

Mouth inferior. Rostral fold developed, pendulous, and margin entirely crenulated. Prominent papillae densely covering margin of rostral fold and anterior part of lower lip. Rostral fold covering upper jaw and connected with lower lip around corner of mouth. Upper lip reduced to thin membrane. Lower lip partitioned into one median lobe and two lateral lobes by long postlabial grooves, with median lobe wider than one-third of mouth gape. Lower lip separated from lower jaw by deep groove. Tiny keratinized tubercles present on snout tip, and slightly more obvious in males than females. Two pairs of long barbels. Rostral barbels reaching posteriorly beyond anterior margin of eyes; maxillary barbels reaching posteriorly beyond posterior margin of eyes.

Dorsal fin with 3 soft unbranched rays and 8 (6) branched rays, origin nearer to tip of snout than to caudal-fin base; margin of fin concave. Anal fin with 3 unbranched and 5 (6) branched rays, margin concave, posterior tip not reaching caudal-fin base, but exceeding midway between bases of anal and caudal fins. Pectoral fin with 12 (1), 13 (1), 14 (2), or **15** (2) branched rays, extending posteriorly midway between pectoral- and pelvic-fin origins. Pelvic fin with 8 (2)–**9** (4) branched rays, origin posterior to dorsal-fin origin; tip not reaching anal-fin origin, extending posteriorly midway between pelvic- and anal-fin origins. Anus very close to anal-fin origin; distance from anus to anal-fin less than one eye diameter. Caudal fin with 9 + 8 (6) branched rays, forked, upper lobe slightly longer than lower lobe.

**Fig 6:**
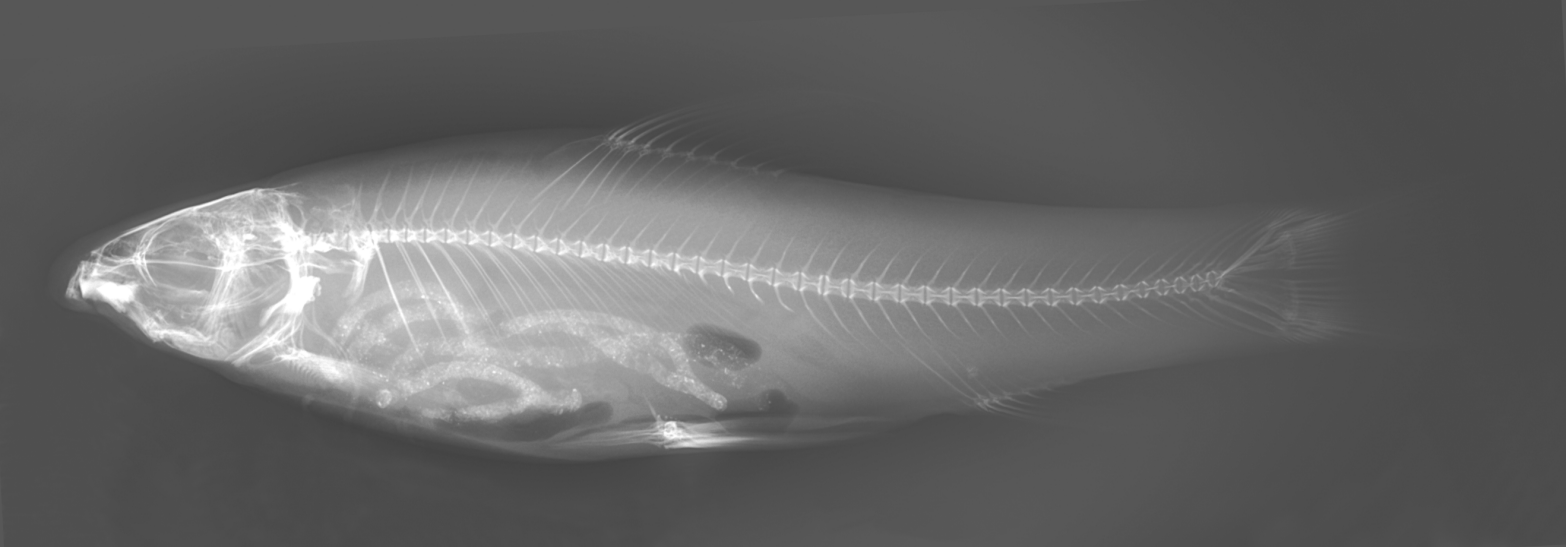
Radiograph of *Zuojiangia jingxiensis*, paratype, KIZ 2012005694, 93.6 mm SL.

Scales large. Pre-dorsal midline scales smaller than flank scales, not embedded under skin. Lateral line complete and straight, ending at caudal-peduncle base; 43 (1), 44 (1), **45** (3), or 46 (1) lateral line scales; 6 (6) scale rows between lateral line and dorsal-fin origin; 5 (6) scale rows between lateral line and pelvic-fin origin; 16 (3) –**18** (3) circumpeduncular-scale rows. **42**–44 [**23+19** (4)– 24+20 (1)] vertebrae (Fig 6).

#### Color in preservative

Body dark brown dorsally and laterally, light brown ventrally. Dark stripe along lateral line on flank, ending at caudal-fin base, width equal to two scales. Fin rays black, membrane hyaline.

#### Color in life

**Fig 7:**
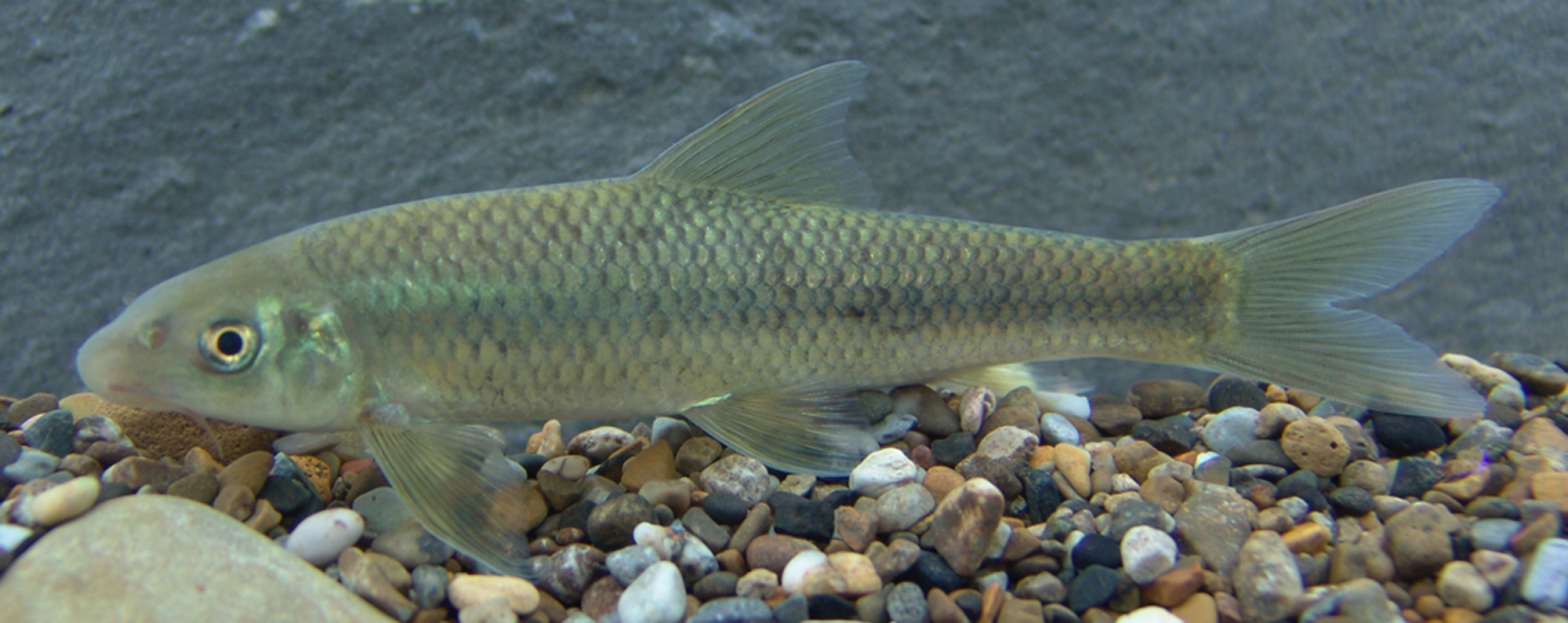
*Zuojiangia jingxiensis*, alive, not preserved, about 100 mm SL, China: Guangxi: Pearl River.

Similar to pattern described for specimens preserved in preservative, but with dark green color, and stripe more obvious (Fig 7).

#### Etymology

Named for Jingxi, where the type species was discovered. Treated as a noun in apposition.

#### Distribution

*Zuojiangia jingxiensis* is presently known only from a karst cave outlet and small connected unnamed stream, which is a tributary of Zuojiang River, in Jingxi County, Guangxi Province, China, draining into the Pearl River.

#### Habitat

*Zuojiangia jingxiensis* lived in a karst cave outlet and connected stream, 1–2 m in depth, sympatric with *Balitora ludongensis*, *Cophecheilus bamen*, *Prolixicheilus longisulcus*, *Rectoris longibarbus* and *Yunnanilus jinxiensis*.

## Discussion

The phylogeny of Labeonini has been demonstrated gradually by recent studies [1–3, 20]. Yang et al. [1] discerned four main clades for Labeonini, that is, the Labeoina, Garraina, Osteochilina, and Semilabeoina subtribes. *Labeo*, *Cirrhinus*, and *Bangana* from South and Southeast Asia form the first clade, and are characterized by the nearly smooth margin of the rostral fold (vs. crenulated in *Zuojiangia*) and developed upper lip (vs. vestigial in *Zuojiangia*). The second clade is represented by *Paracrossochilus*, *Tariqilabeo*, and the species of *Garra* from Africa, Southeast Asia, and South Asia. *Paracrossochilus* has papillose lateral lobes of the upper lip (vs. vestigial in *Zuojiangia*), but absent or vestigial on the median part. *Tariqilabeo* has a very thin (sometimes absent) upper lip that widens at the corner of the mouth (vs. absent in *Zuojiangia*) [21, 22]. *Garra* is characterized by having a disc on the lower lip (vs. absent in *Zuojiangia*). The third clade includes most species distributed in Southeast Asia, but also found in southwestern China: *Epalzeorhynchos*, *Crossocheilus*, *Henicorhynchus*, *Lobocheilos*, *Barbichthys*, *Cirrhinus molitorella* (from China), *Thynnichthys*, *Labiobarbus*, and *Osteochilus*. *Epalzeorhynchos* has movable rostral lobes dorsal to the rostral barbels (vs. absent in *Zuojiangia*). *Crossocheilus*, *Henicorhynchus*, *Lobocheilos*, *Barbichthys*, *Cirrhinus molitorella*, *Labiobarbus*, and *Osteochilus* all have a developed upper lip (vs. vestigial in *Zuojiangia*) [21]. *Thynnichthys* (type species: *Thynnichthys thynnoides* Bleeker 1852) has a unique mouth morphology different from all other labeonins, and only a thin rostral fold, simple lower lip, no upper lip, no barbels, and wide and terminal mouth [1]. The genera and species included in the fourth clade are mainly endemic to China. Zheng et al. [2] focused on the phylogeny of the genera and species of Labeonini endemic to China. Within this clade, *Mekongina* has a fimbriate margin of the rostral fold (vs. crenulated in *Zuojiangia*), and long mental grooves (vs. absent in *Zuojiangia*), partitioning the lower lip into three parts. Of species of *Garra* from China, *Placocheilus* and *Sinigarra* have a disc with a central fleshy pad on the lower lip (vs. absent in *Zuojiangia*), and two rows of pharyngeal teeth (vs. three in *Zuojiangia*). The species of *Bangana* from China have a nearly smooth margin of the rostral fold (vs. crenulated in *Zuojiangia*) and developed upper lip (vs. vestigial in *Zuojiangia*). The remaining genera can be further divided into four sublineages according to Zheng et al. [2]. The first sublineage includes *Cophecheilus* and *Prolixicheilus*, which share the long postlabial grooves and non-split rostral fold. The second sublineage includes the species of *Parasinilabeo*. *Zuojiangia* is distinguished from *Parasinilabeo* (except for *Parasinilabeo longibarbus*) by having long postlabial grooves (vs. short, at corners of the mouth), arched rostral fold, with an entirely crenulated margin (vs. crescent rostral fold with vertical splits), and long maxillary barbels, reaching posteriorly beyond the posterior margin of the eyes (vs. short, not reaching). *Pseudocrossocheilus*, *Ptychidio*, *Rectoris*, *Semilabeo*, and *Stenorynchoacrum* are included within the third sublineage. The new genus differs from *Pseudocrossocheilus*, *Ptychidio*, *Rectoris*, and *Semilabeo* by having long postlabial grooves (vs. short, only at corners of the mouth), arched rostral fold, with an entirely crenulated margin (vs. long and bifurcate tassels in *Ptychidio*; fimbriate in *Pseudocrossocheilus* and *Rectoris*; and smooth in *Semilabeo*), the transverse branch of the dentary longer than half the length of the longitudinal branch (vs. two-thirds in *Rectoris*); the premaxilla bears the obvious ascending process (vs. lacks in *Rectoris*); and from *Pseudocrossocheilus* in having no mental grooves (vs. long mental grooves, partitioning lower lip into three parts). *Stenorynchoacrum* differs from the new genus by having an undeveloped rostral fold in the middle part and everted on two sides of the rostral fold (vs. pendulous in *Zuojiangia*). The fourth sublineage consists of *Discocheilus*, *Discogobio*, *Hongshuia*, *Sinocrossocheilus*, *Paraqianlabeo*, and *Pseudogyrinocheilus*. *Discocheilus* and *Discogobio* have a disc with a central fleshy pad on the lower lip (vs. absent in *Zuojiangia*). *Hongshuia* and *Sinocrossocheilus* have a fleshy pad on the lower lip (vs. absent in *Zuojiangia*), two rows of pharyngeal teeth (vs. three in *Zuojiangia*), and seven branched dorsal-fin rays (vs. eight in *Zuojiangia*). *Paraqianlabeo* has a suctorial lower lip (vs. absent in *Zuojiangia*), short postlabial grooves (vs. long in *Zuojiangia*), and mental grooves (vs. absent in *Zuojiangia*). *Pseudogyrinocheilus* has short postlabial grooves (vs. long in *Zuojiangia*), smooth rostral fold margin (vs. crenulated in *Zuojiangia*), and everted rostral fold (vs. pendulous in *Zuojiangia*). Thus, these genera are easily distinguished from the new genus.

*Zuojiangia* resembles *Cophecheilus*, *Prolixicheilus*, and *P. longibarbus* within Labeonini, but can be easily distinguished from these three genera based on morphological characters. In addition to the oral morphological characters, *Zuojiangia* largely differs from *Cophecheilus*, *Prolixicheilus*, and *P. longibarbus* by the osteological characters of the head and jaw. In the new genus, the dentary in the lower jaw is shaped like an inverted L in ventral view (Fig 3C), the transverse branch is longer than half the length of the longitudinal branch (vs. shorter in *Cophecheilus*, *Prolixicheilus*, and *P*. *longibarbus*) (Fig 3G, 3K and 3O). A stubby lateral process is present at the anterolateral margin of the longitudinal branch (Fig 3C, lp), close to the corner (vs. at the midpoint of the longitudinal branch in *Cophecheilus* (Fig 3G); no lateral process in *Prolixicheilus* (Fig 3K); and at one third the anterior position of the longitudinal branch in *P*. *longibarbus* (Fig 3O)). In the upper jaw, the premaxilla bears a triangular ascending process tapering to a point (Fig 3D) (vs. slenderer in *Cophecheilus* (Fig 3H); dominant, broad with a blunt tip in *Prolixicheilus* (Fig 3L); and lacks an obvious ascending process in *P*. *longibarbus* (Fig 3P)). The maxilla exhibits a pair of articular heads at the anterodorsal margin, and a distinct fingerlike descending process posterior to the medial articular head embracing the ascending process of the premaxilla (Fig 3D, fp mx) (vs. fingerlike process of the maxilla vestigial in *Cophecheilus* (Fig 3H); well-developed and embracing the ascending process of the premaxilla (invisible in the anterior view) in *Prolixicheilus* (Fig 3L); and well-developed and articular head of the maxilla rudimentary in *P*. *longibarbus* (Fig 3P)).

**Fig 8:**
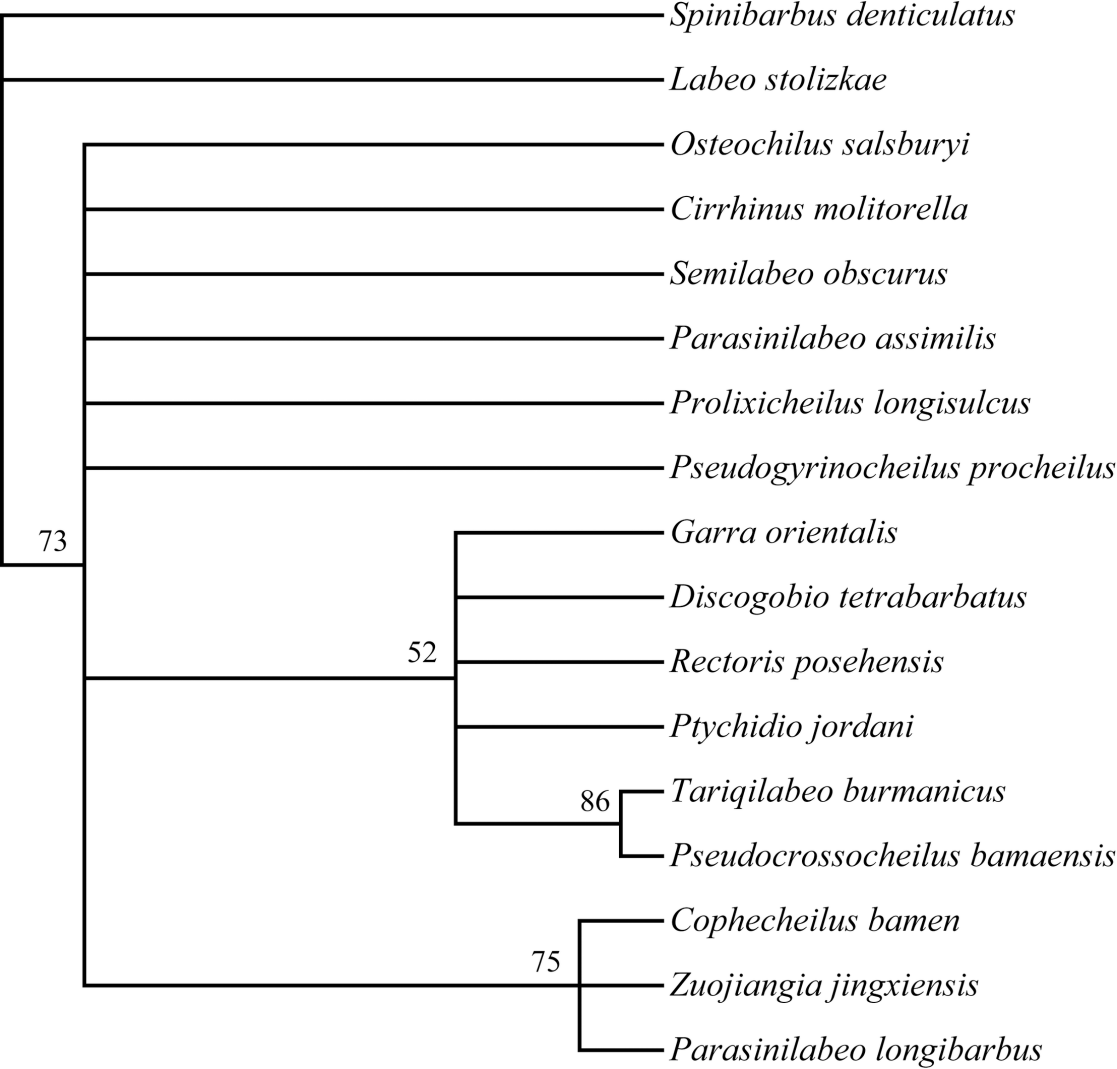
Maximum likelihood tree of Labeonini based on thirteen morphological characters. The nodal numbers are bootstrap values (1000 replicates), and only values above 50% are given.

Thirteen morphological characters are used to construct the phylogenetic tree (S2 Table). The morphological phylogenetic analysis indicated that the new genus is closely related to *Cophecheilus bamen* and *Parasinilabeo longibarbus* (Fig 8). The molecular results suggested that *Cophecheilus* and *P. longibarbus* were located at the base of the karst group of Labeonini [2]. Based on our morphological analysis and previous molecular results, it is conceivable that the phylogenetic position of *Zuojiangia* may also be located at the base of the karst group of Labeonini, showing relatively close relationships with *Cophecheilus*and *P. longibarbus*.

The specimens of the new genus were preserved in formalin after their collection, and unfortunately the type locality was destroyed in 2013. Therefore, molecular tissue and new specimens from the type locality are no longer available for study. Because its known habitat has been destroyed, the new genus can be considered rare and likely endangered. To identify the evolutionarily significant unit and protect this rare species, it is necessary to describe the new genus as soon as possible. This work is part of our study on the Labeonini in Southwest China. We hope that the discovery and description of new genera and species will arouse social concern and conservation awareness.

## Comparative materials

*Cophecheilus bamen*: KIZ 20150001, 003, 004, 3 ex., Jingxi, Guangxi, China.

*Cophecheilus brevibarbatus*: KIZ 20140717001–007, 7 ex., Tiandong, Guangxi, China.

*Discocheilus wuluoheensis*: KIZ 20080384–387, 20080428–429, 6 ex., Luoping, Yunnan, China.

*Discogobio yunnanensis*: KIZ 20080437–456, 20 ex., Zunyi, Guizhou, China.

*Garra imberba*: KIZ 2008021–025, 065–076, 17 ex., Xishuangbanna, Yunnan, China.

*Hongshuia megalophthalmus*: KIZ 2008005469–470, 480, 484, 487, 488, 494, 496, 497, 502, 503, 505, 507, 509, 520, 15 ex., Tian’e, Guangxi, China.

*Hongshuia microstomatus*: KIZ 2008005150–152, 2008005154–161, 163, 164, 167, 168, 15 ex., Nandan, Guangxi, China.

*Mekongina lancangensis*: KIZ 96060478, 043, 041, 458, 459, 5 ex., Mengla, Yunnan, China.

*Parasinilabeo longibarbus*: KIZ 20100005–015, 11 ex., Fuchuan, Guangxi, China.

*Parasinilabeo assimilis*: KIZ 20080093, 1 ex., Yongfu; 20110247–249, 3 ex., Lipu, Guangxi, China.

*Placocheilus caudofasciatus*: KIZ 200401009–012, 4 ex., Lvchun, Yunnan, China.

*Prolixicheilus longisulcus*, KIZ 2008007432–7437, 454–458, 11 ex., KIZ 20100063–065, 3 ex., Jingxi, Guangxi, China.

*Pseudocrossocheilus bamaensis*: KIZ 2001000019–0021, 024, 040, 044, 045, 054, 055, 078, 095–097, 103, 104, 15 ex., Du’an, Guangxi, China.

*Pseudogyrinocheilus prochilus*: 2006007035–039, 5 ex., Zhanyi, Yunnan, China.

*Rectoris posehensis*: KIZ 20100016–021, 6 ex., Shanglin, Guangxi, China.

*Semilabeo obscurus*: KIZ 2008084–085, 2 ex., Libo, Guizhou; KIZ 20100052, 1 ex., Bama, Guangxi, China.

*Sinigarra napoensis*: KIZ 20130170–172, 3 ex., Napo, Guangxi, China.

*Sinocrossocheilus labiata*: KIZ 995325, 327–331, 334–336, 338–342, 344–346, 18 ex., Tongzi, Guizhou, China.

*Stenorynchoacrum xijiangensis*: KIZ 2001060482–488, 490−494, 12 ex., Guilin, Guangxi, China.

*Tariqilabeo burmanicus*: KIZ 20050415006–009, 4 ex., Gengma, Yunnan, China.

*Tariqilabeo bicornis*: KIZ 20041114003–008, 6 ex., Baoshan, Yunnan, China.

## Supporting information

S1 Table Raw measurement data. (XLSX)

S2 Table Matrix of the morphological characters used for morphological phylogenetic analysis. (XLSX)
